# Proposal of a Two-Tier System in Grouping Adenocarcinoma of the Uterine Cervix

**DOI:** 10.3390/cancers12051251

**Published:** 2020-05-15

**Authors:** Hiroko Machida, Koji Matsuo, Shinya Matsuzaki, Wataru Yamagami, Yasuhiko Ebina, Yoichi Kobayashi, Tsutomu Tabata, Masanori Kaneuchi, Satoru Nagase, Takayuki Enomoto, Mikio Mikami

**Affiliations:** 1 Department of Obstetrics and Gynecology, Tokai University School of Medicine, Kanagawa 259-1193, Japan; hiroko.machida@tokai.ac.jp; 2 Division of Gynecologic Oncology, Department of Obstetrics and Gynecology, University of Southern California, Los Angeles, CA 90089, USA; Koji.Matsuo@med.usc.edu; 3 Norris Comprehensive Cancer Center, University of Southern California, Los Angeles, CA 90089, USA; 4 Department of Obstetrics and Gynecology, Osaka University Graduate School of Medicine, Osaka 565-0871, Japan; zacky_s@gyne.med.osaka-u.ac.jp; 5 Department of Obstetrics and Gynecology, Keio University School of Medicine, Tokyo 160-8582, Japan; gami@z8.keio.jp; 6 Department of Obstetrics and Gynecology, Kobe University Graduate School of Medicine, Kobe 650-0017, Japan; ebiyas@med.kobe-u.ac.jp; 7 Department of Obstetrics and Gynecology, Kyorin University School of Medicine, Tokyo 181-8611, Japan; yoichi@ks.kyorin-u.ac.jp; 8 Department of Obstetrics and Gynecology, Tokyo Women’s Medical University, Tokyo 162-8666, Japan; tabata.tsutomu@twmu.ac.jp; 9 Department of Obstetrics and Gynecology, Otaru General Hospital, Hokkaido 047-8550, Japan; kaneuchi-ma@otaru-general-hospital.jp; 10 Department of Obstetrics and Gynecology, Yamagata University, Yamagata 990-9585, Japan; nagases@med.id.yamagata-u.ac.jp; 11 Department of Obstetrics and Gynecology, Niigata University School of Medicine, Niigata 951-8510, Japan; enomoto@med.niigata-u.ac.jp

**Keywords:** cervical cancer, adenocarcinoma, histological subtypes, survival

## Abstract

(1) *Background*: This study examined the use of a two-tier system in grouping cervical adenocarcinoma for survival discrimination. (2) *Methods*: A nationwide retrospective observational cohort study was conducted using the Japan Society of Gynecologic Oncology tumor registry database from 2001 to 2015 (*n* = 86,754). Adenocarcinoma subtypes were grouped as type 1 (endocervical usual type and endometrioid) or type 2 (serous, clear, mucinous, and not otherwise specified), based on their relative survival compared with that of squamous tumors. (3) *Results*: The majority of the adenocarcinoma cases were type 1 (*n* = 10,121) versus type 2 tumors (*n* = 5157). Type 2 tumors were more likely to be old and have stage IV disease than those with squamous tumors. The number of type 2 tumors increased from 2001 to 2014 (106.1% relative increase, *p* < 0.001). Type 2 tumors had disproportionally poorer survival compared to other types (5-year survival rates: 68.9% for type 2, 75.4% for type 1, and 78.0% for squamous; *p* < 0.001). On multivariate analysis, type 2 tumors remained an independent prognostic factor associated with decreased survival compared with squamous (adjusted hazard ratio 2.00, 95% CI 1.84–2.15, *p* < 0.001). (4) *Conclusion*: The survival of cervical adenocarcinoma varies largely across the histological subtypes, and the proposed two-tier grouping may be useful for survival discrimination.

## 1. Introduction

Cervical cancer is one of the most common gynecologic malignancies in Japan, and approximately 10,000 women were newly diagnosed with invasive cervical cancer in 2019 [1]. Recently, the age-adjusted incidence rate of cervical cancer increased in Japan from 4.5 to 11.9 per 100,000 women between 1975 and 2015, whereas that in the United States decreased from 16.3 to 11.4 per 100,000 women in this period [2]. Particularly, the proportion of adenocarcinomas in cervical cancer cases in Japan increased between 1976 and 2012 [2,3]. The increased incidence of cervical cancer in Japan can be partially explained by the extremely low cervical cancer screening rate, estimated at approximately 40% of the population, and the fact that the oncogenic human papillomavirus (HPV) vaccination program is currently suspended per the Ministry of Health, Labor and Welfare [1].

Cervical cancer comprises various histologic types, and the most common histological subtypes are squamous, adenocarcinoma, and adenosquamous carcinoma [4,5]. The histological subtype has been recognized as one of the important prognostic factors in cervical cancer. Previous studies in the United States and Japan have shown that women with cervical adenocarcinoma have poorer survival compared to those with squamous tumors [6,7,8]. Despite this, the survival outcome of different histological subtypes has not been completely studied.

Little has been studied about cervical adenocarcinomas previously, mainly because most epidemiologic studies lacked sufficient cases to separately study these less common tumors. Recent years have witnessed major progress in understanding the molecular biology of cervical cancer [5], but it has not yet been translated into individualized treatment for these women or improvements in their disease outcome. Instead, there continues to be a “one-size-fits-all” approach with regard to prognosis and treatment. Here, the present study proposes a two-tier system in grouping cervical adenocarcinomas for survival discrimination.

The primary objective of our study was to propose a two-tier system for grouping cervical adenocarcinoma and to identify characteristics of type 1 and type 2 cervical adenocarcinoma compared to squamous cell carcinoma (SCC). The secondary objective was to examine the utility of the two-tier system in grouping cervical adenocarcinoma for survival discrimination.

## 2. Materials and Methods

### 2.1. Data

This was a society-based retrospective observational study that used the Japan Society of Obstetrics and Gynecology (JSOG) tumor registry database. This nationwide project was conducted by the Japan Society of Gynecologic oncology (JSGO); the dataset was provided by the Gynecologic Tumor Committee of JSOG, and the research functioned as a JSGO–JSOG joint study [9]. The JSOG database is a cancer registry for gynecologic malignancy that records comprehensive information for cancer types, tumor characteristics, treatment types, and survival outcomes between 2001 and 2015. The registry is maintained by the Gynecologic Tumor Committee of JSOG and comprises 466 local and leading regional hospitals that cover approximately half (55.6%) of all new patients with gynecological malignancy in Japan [10,11]. The JSOG Ethics Committee (2018-36-67) and the Institutional Review Board of the hosting institution, Tokai University School of Medicine (17R-100), approved this study. Each participating institution reviewed the protocol and approved as appropriate.

### 2.2. Eligibility Criteria

Women with invasive cervical cancer of one of the three major histology types (squamous, adenocarcinoma, or adenosquamous carcinoma) who underwent initial treatment from 2001 to 2015 were included. Women with other rare adenocarcinoma subtypes, sarcoma, a metastatic tumor from another origin, or tumors of unknown histology (including unspecified adenocarcinoma), unknown stage, or unknown age were excluded.

### 2.3. Clinical Information

Patient demographics, tumor characteristics, and treatment types were abstracted from the database. Patient demographics included age (<40, 40–49, 50–59, 60–69, and ≥70 years), year (2001–2005, 2006–2010, and 2011–2015), and registry area (East, Central, West, and North), as previously defined [11]. Tumor characteristics included histological type, cancer stage, and lymph node involvement (yes versus no). Initial treatment types included surgical management, concurrent chemoradiotherapy, radiotherapy alone, chemotherapy alone, and others. Survival outcomes included follow-up time, vital status, and cause of death.

### 2.4. Study Definition

The recorded cancer stage was classified based on the 2008 International Federation of Gynecology and Obstetrics (FIGO) [12]. Cause-specific survival (CSS) was defined as the time interval between cervical cancer diagnosis and death from cervical cancer. Cases without a survival event or those that were lost to follow-up were censored at the last visit with a known vital status.

### 2.5. External Validation Cohort

The validation cohort used the Surveillance, Epidemiology, and End Results (SEER) program. The SEER registry is a population-based database launched in 1973 that is supported and managed by the National Cancer Institute in the United States. The most recent registry covers approximately 34.6% of the US population from 11 states and seven areas. SEER registries collect data on patient demographics, tumor characteristics, treatment types, and survival outcomes. In this study, SEER cases over the same period were abstracted from the database using SEER*Stat 8.3.5 (IMS Inc., Calverton, MD, USA) [13]. The International Classification of Diseases for Oncology revision 3 site/histology validation list and the WHO histological classification were used for grouping the histological subtypes, based on a prior study [4].

### 2.6. Population Trend Estimation

Based on the JSOG data and Cancer Registry and Statistics data for cervical cancer cases registered in the study period [14], the age-standardized incidence rate was calculated per 100,000 population. The age-standardized incidence rate was adjusted using the population pyramid for 1985 as the standard population (model population), as described in a prior study [2]. This study used the Joinpoint Regression Program 4.8.0.0 and showed the Annual Percent Change (APC) of age-adjusted incidence rates in the histological subtypes of cervical cancer. Linear segmented regression analysis was utilized for the model, and log transformation of the data was performed to determine the annual percentage change in the slope along with a 95% confidence interval.

### 2.7. Statistical Analysis

Continuous variables were expressed as the mean and standard deviation or the median and interquartile range (IQR), as appropriate, and statistical differences were assessed using Student’s *t-*test or the Mann–Whitney *U* test. For categorical or ordinal variables, statistical differences were assessed with the chi-square test or Fisher exact test, as appropriate. A binary logistic regression model was fitted to identify the independent clinicopathological factors associated with type 2 adenocarcinoma for multivariable analysis. The age, year, and registry area, as well as hysterectomy and lymphadenectomy data and histology type, were entered in the final model, and the effect size was expressed as odds ratio (OR) and 95% confidence interval (CI). The Hosmer–Lemeshow test was used to assess the goodness of fit, and *p* > 0.05 was interpreted as a good model.

The Kaplan–Meier method was used to construct the survival curves, and the difference between the curves was assessed using the log-rank test. Association of histology type and CSS was adjusted for age, year at diagnosis, registry area, cancer stage, nodal involvement, and initial treatment in multivariable analysis. The Cox proportional hazard regression model was used for the analysis, and the effect size was expressed as a hazard ratio (HR) and 95% CI. At the time of analysis, survival data were matured and available for cases between 2001 and 2011.

A sensitivity analysis was undertaken to examine the robustness of the study results. External validation was performed using the SEER program, as above. All statistical analyses were based on a two-sided hypothesis, and *p* < 0.05 was considered statistically significant. SPSS (version 25.0, Armonk, NY, USA) was used for all the analyses. The Strengthening the Reporting of Observational Studies in Epidemiology (STROBE) guidelines were consulted to display the results of the observational cohort study [15].

### 2.8. Ethical Committee Approval

Japan Society of Obstetrics and Gynecology (2018-36-67), and Tokai ethical committee (17R-100).

## 3. Results

The study-specific proposal for the two-tier system in grouping cervical adenocarcinomas is shown in Figure S1A1. The crude survival curves for CSS were plotted for each of the histological subtypes of adenocarcinoma (Figure S1A1,B). Adenocarcinoma classification by the World Health Organization (WHO) was compared with that by the International Endocervical Criteria and Classification (IECC) (Table S1). This study grouped the histology types to a more favorable group (type 1 adenocarcinoma) and a less favorable group (type 2 adenocarcinoma) based on the cluster patterns for adenocarcinoma type relative to survival in the squamous group. Among adenocarcinomas, adenocarcinoma not otherwise specified (NOS) included type 2 adenocarcinoma because it represented one-fourth of all adenocarcinomas and exhibited similar cluster patterns to other type 2 adenocarcinoma subtypes (Table S2, Figure S1A2). Therefore, type 1 adenocarcinoma includes endocervical adenocarcinoma usual type and endometrioid. Type 2 was defined as serous, mucinous, clear cell, and NOS histology subtypes.

### 3.1. Patient Demographics

**Figure 1:**
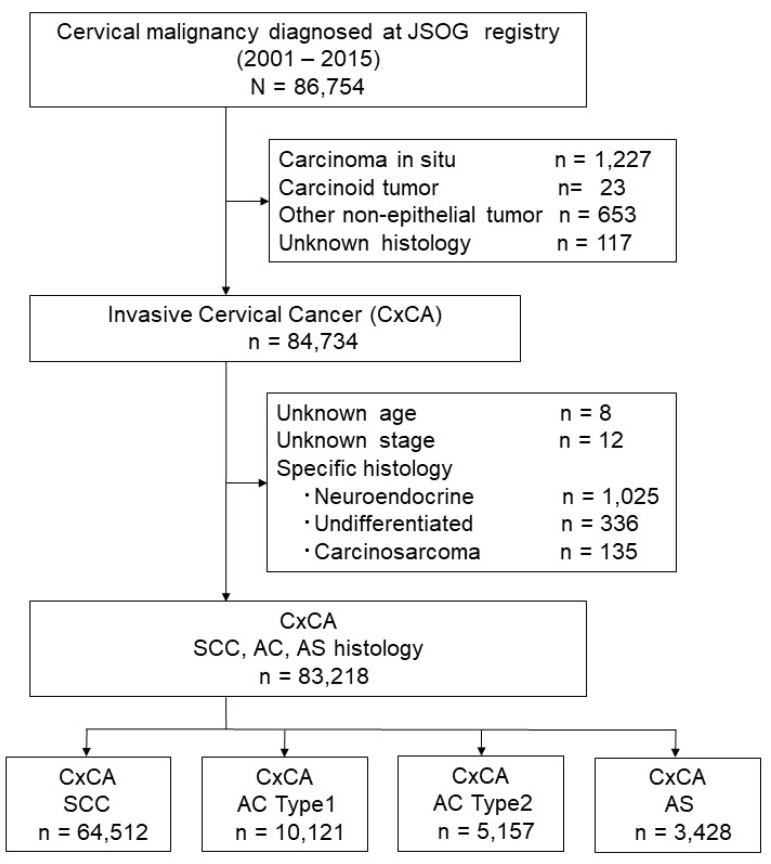
Schema for patient selection.

The patient selection schema is shown in Figure 1. During the study period, 86,754 cases of women with cervical malignancies were recorded in the JSOG tumor registry. The final study population comprised 83,218 women with the three major histological subtypes of invasive cervical cancer. The most common histology type was squamous cell carcinoma (SCC) (*n* = 64,512; 77.5%) followed by adenocarcinoma (*n* = 15,278; 18.4%) and adenosquamous carcinoma (*n* = 3428; 4.1%). Among adenocarcinomas, the majority were type 1 (*n* = 10,121; 66.2%) versus type 2 (*n* = 5157; 33.8%).

Demographics of each of the histology types are described in Table 1. In the entire cohort, women with type 2 adenocarcinoma were more likely to be older at diagnosis (median 54 versus 46–52 years, *p* < 0.001), be registered in the Northern regions of Japan, have recent years of diagnosis, and have a tumor with stage IV compared to those with other histological subtypes (all, *p* < 0.001). In the multivariable analysis (Table S3), an older age at diagnosis, recent diagnosis, and stage IV disease remained independent factors for type 2 adenocarcinoma (all, *p* < 0.001).

Among women with adenocarcinomas (Table S2), those with type 2 adenocarcinoma, especially mucinous, serous, and clear cell subtypes, were more likely to be older, to be registered in the Northern regions of Japan, and to have more recent years of diagnosis compared to those with type 1 adenocarcinoma (all, *p* < 0.001). In the multivariable analysis (Table S4), an older age at diagnosis, recent diagnosis, stage IV disease, and non-surgical treatment remained independent factors for type 2 adenocarcinoma (all, *p* < 0.001).

### 3.2. Histological Type-Specific Trends

**Figure 2:**
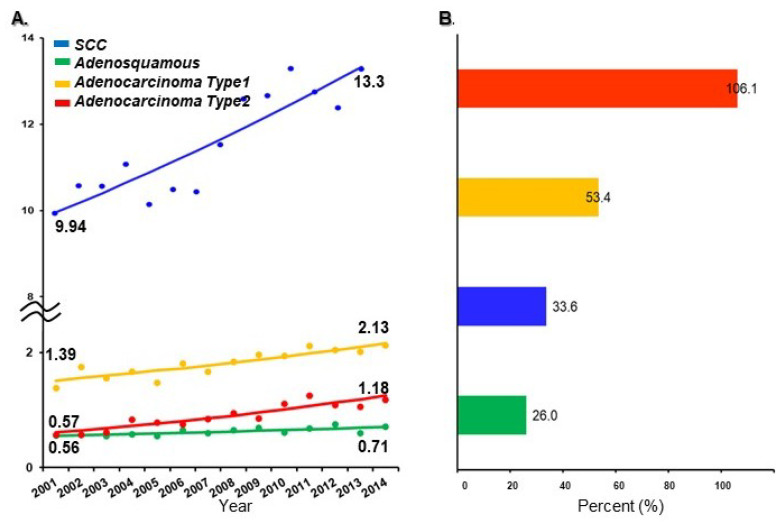
Age-adjusted incidence rate and relative change in histological subtypes. (**A**) Age-adjusted incidence rate in histological subtypes per 100,000 population (2001–2015, cervical cancer). Age-adjusted incidence rate of cervical cancer was analyzed using the Japanese model population for 1985. (**B**) Relative change increase per histological subtype.

The temporal trends of type 2 adenocarcinoma histology per 100,000 population were examined using the JSOG database and the National Cancer Center Database in Japan (Figure 2A). The number of type 2 adenocarcinoma cases significantly increased (106.1% relative increase, APC 5.62, 95% CI 3.91–7.36, *p* < 0.001) such that the interval increase was higher compared to type 1 adenocarcinoma (53.4% relative increase, APC 2.75, 95% CI 1.79–3.72, *p* < 0.001), SCC (33.6% relative increase, APC 2.28, 95% CI 1.54–3.01, *p* = 0.001) and adenosquamous carcinoma (26.0% relative increase, APC 1.97, 95% CI 0.99–2.97, *p* = 0.07) cases between 2001 and 2014 (Figure 2B).

### 3.3. Cause-Specific Survival for Histological Subtypes 

**Figure 3:**
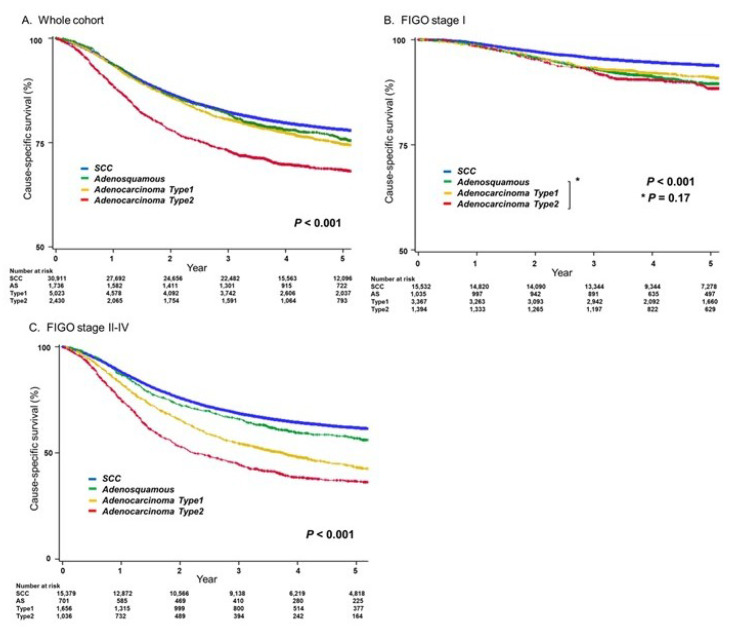
Cause-specific survival for histological subtypes. Log-rank test for *p*-value. The Y-axis was truncated to 50–100% for panels **A**–**B** and 0–100% for panel C. Survival curves were constructed for the cause-specific survival of women (panel A), women with stage I (panel B), and women with stage II–IV (panel **C**). Abbreviations: SCC, squamous cell carcinoma.

Survival analyses were assessed in 41,717 women diagnosed between 2002 and 2011. The median follow-up time was 3.95 (IQR, 2.37–5.41) years, and there were 8522 deaths from cervical cancer during follow-up. In univariate analysis, women with type 2 adenocarcinoma had significantly lower CSS compared to those with SCC (5-year rates, 68.9% versus 78.0%; *p* < 0.001; Figure 3A). The survival was assessed in women with stage I disease (*n* = 22,146); type 2 adenocarcinoma had significantly lower CSS compared to SCC (5-year rates, 88.3% versus 94.0%; *p* < 0.001; Figure 3B). However, the 5-year CSS rate of type 2 adenocarcinoma was similar to that of type 1 adenocarcinoma or adenosquamous carcinoma (89.4–91.0%, *p* = 0.17). Among 19,571 women with stage II–IV disease, women with type 2 adenocarcinoma had the worst CSS of all the histological subgroups (5-year rates, 36.7% for type 2 adenocarcinoma, 43.5% for type 1 adenocarcinoma, 56.8% for adenosquamous carcinoma, and 61.8% for squamous carcinoma, *p* < 0.001; Figure 3C).

Multivariate analysis was performed for CSS (Table 2). After controlling for age, year of diagnosis, registry area, cancer stage, and initial treatment, type 2 adenocarcinoma demonstrated the largest impact on CSS (adjusted-HR 2.00, 95% CI 1.84–2.15) followed by type 1 adenocarcinoma (adjusted-HR 1.95, 95% CI 1.82–2.08) and adenosquamous carcinoma (adjusted-HR 1.50, 95% CI 1.35–1.66) compared to squamous carcinoma (all, *p* < 0.001).

### 3.4. SEER Registry Database

Similar results were observed in the external validation cohort (SEER dataset, *n* = 39,966). Among the whole cohort, there were 1658 (4.1%) women with type 2 adenocarcinoma (Table S5). Women with type 2 adenocarcinoma were more likely to have an older age at diagnosis, to be of Asian ethnicity, to be registered in the western region, and to be diagnosed with stage IV disease compared to those with other histological subtypes (all, *p* < 0.001). 

The median follow-up time was 4.1 (IQR, 1.5–9.0) years, and there were 11,439 deaths from cervical cancer during follow-up. In multivariate analysis, women with type 2 adenocarcinoma had significantly lower CSS than those with SCC (5-year rates, 65.7% versus 69.8%; adjusted-HR 1.45, 95% CI 1.33–1.58; *p* < 0.001; Figure S2A). The survival was assessed in women with stage I disease, type 2 adenocarcinoma had significantly lower CSS compared to SCC (5-year rates, 89.5% versus 91.6%; p < 0.002; Figure S2B). Women with stage IIB–IVA disease, who had type 2 adenocarcinoma histology, had significantly lower CSS compared to those with other histology types (5-year rates, type 2 versus others, 41.5% vs. 48.0–59.9%; *p* < 0.001; Figure S2C).

## 4. Discussion

The present study demonstrates that the survival of cervical adenocarcinoma varies largely across the histological subtypes, and tumors with type 2 adenocarcinoma histology are associated with worse CSS. The present study proposes a two-tier grouping system, which may be useful for survival discrimination and is effective for describing the different characteristics of cervical adenocarcinoma subtypes. Additionally, type 2 adenocarcinoma had aggressive tumor characteristics and poor prognosis, and its incidence has increased significantly in recent years in Japan.

Our study captures the characteristics and trends for the histological variants of cervical adenocarcinoma in Japan and clearly demonstrates that cervical adenocarcinoma is not a single disease entity. On the basis of differences in survival and clinical outcomes, cervical adenocarcinoma can be divided into two types. These differences could be partly explained by differences in HPV-related factors [16,17] as well as socioenvironmental and genetic factors [5].

Type 2 adenocarcinoma was more frequently observed in older women in our study, and they are usually not associated with high-risk HPV, unlike SCC [18]. These histological subtypes have unique histological and molecular features. Mucinous tumors, including gastric and intestinal types, are reported to represent as many as 25% of all adenocarcinomas in Japan [19,20]. The genesis of such tumors more closely resembles gastric and intestinal carcinogenesis. p53 mutations are often found in these tumors, and p53 function declines with age, which might contribute to an enhanced mutation frequency, tumorigenesis, and tumor metastasis [21]. Serous tumors arising in older patients are known to be associated with mutant p53 expression [22]. These tumors commonly arise in atrophic epithelial lesions on the endocervical surface and in endocervical glands, and they display aggressive tumor features compared with the findings for endometrioid tumors. Clear cell tumors are rare, and they have developed in two distinct populations in association with in utero diethylstilbestrol (DES) exposure as well as sporadically [23]. DES-exposed patients are rarely identified in Japan, but the incidence of clear cell carcinomas among gynecological malignancies in Japan is substantially increasing because of socioenvironmental factors, such as delays in the age at first menarche and menopause and a lower use of oral contraceptives [24,25]. Additionally, clear cell tumors are known to have chemoresistant characteristics [26], and they may not respond to conventional therapies, resulting in dismal survival.

The two-tier system for grouping cervical adenocarcinoma in our study emphasizes that clinicians should focus on high-risk patients, but further investigation is needed to confirm our findings. Therefore, we are planning a molecular-based study to examine if there is any distinct genetic alteration in type 1 and type 2 tumors. Increased understanding of the molecular heterogeneity that is intrinsic to the various subtypes of cervical cancer will likely shape the future of cervical cancer diagnosis, prognosis, and treatment. 

This study recorded similar survival results for the histological subtypes of cervical adenocarcinoma between the Japanese and American cohorts but also identified differences in the proportions of histological subtypes between these populations. Variations in the histologic subtypes of cervical adenocarcinoma between the Japanese and US cohorts could be partly explained by differences in tumor biology; for example, common HPV genotypes are different between the two countries. [17,27] Variations in the histological subtypes of cervical cancer could be partly explained by differences in HPV-related and epidemiological factors [28,29,30,31]. 

HPV is considered the most important factor in the oncogenesis of cervical cancer. Indeed, previous studies have shown that the prevalence of HPV varies by histological subtype of cervical adenocarcinoma [28,29,31]. Furthermore, HPV is detected in the endocervical usual type and mucinous intestinal tumors but not in rare histological variants of clear cell, mucinous gastric type, or endometrioid and mesonephric tumors. The 2014 WHO classification of cervical tumors categorizes based on morphological features [4,16]. In contrast, the IECC system proposed in 2018 attempts to categorize adenocarcinoma by incorporating the etiology of HPV status (Table S1) [18]. Thus, the present study examined histological variants in cervical adenocarcinoma for survival discrimination using both classifications.

The strengths of the study include the large number of cases. The majority of previous studies have limited sample sizes [32,33], which risk type II errors. By analyzing more than 15,000 cervical adenocarcinoma cases, our study is unlikely to have this problem. Histology-specific analysis provides useful information to clinicians, as even common tumor types display variable differences in characteristics and outcomes. External validation also enhanced the study findings. Our study has some limitations. This was a retrospective study; therefore, there may be confounding factors that could have affected the results. For instance, information regarding the HPV infection status was not available in our study. In addition, the study was retrospective, and there was a lack of a central pathology review; as such, the accuracy of histological subtyping is unknown. The database only focuses on leading hospitals in Japan, such as university hospitals and cancer centers, thereby increasing the possibility of selection bias. 

## 5. Conclusions

In summary, the two-tier system for grouping cervical adenocarcinoma may be useful for survival discrimination and formulation of a new treatment strategy using the morphology profile of cervical adenocarcinoma with poor prognosis. Our analysis of cervical adenocarcinoma subtypes will ultimately address the necessity of developing clinical trials to treat cervical adenocarcinoma patients with distinct therapies. Furthermore, a molecular-based study is warranted to examine if there is any distinct genetic difference in type 1 and type 2 tumors.

## Figures and Tables

**Table 1:** Patient demographics of cervical cancer (*n* = 83,218).

Characteristics	SCC	AC Type 1	AC Type 2	AS	*p*-Value
Number	64,512 (77.5%)	10,121 (12.2%)	5157 (6.2%)	3428 (4.1%)	
Age (years)	52 (40–65)	49 (40–60)	54 (42–65)	46 (39–57)	**<0.001**
<40	15,074 (23.4%)	2326 (23.0%)	973 (18.9%)	960 (28.0%)	
40–49	14,361 (22.3%)	2971 (29.4%)	1321 (25.6%)	1069 (31.2%)	
50–59	12,111 (18.8%)	2282(22.5%)	1031 (20.0%)	699 (20.4%)	
60–69	11,305 (17.5%)	1517 (15.0%)	934 (18.1%)	440 (12.8%)	
≥70	11,661 (18.1%)	1025(10.1%)	898 (17.4%)	260 (7.6%)	
Registry area					**<0.001**
North	4426 (6.9%)	661 (6.5%)	384 (7.4%)	184 (5.4%)	
Central	9475 (14.7%)	1615 (16.0%)	804 (15.6%)	477 (13.9%)	
East	22,590 (35.0%)	3784 (37.4%)	1901 (36.9%)	1476 (43.1%)	
West	28,021 (43.4%)	4061 (40.1%)	2068 (40.1%)	1291 (37.7%)	
Year at diagnosis					**<0.001**
2001–2005	17,638 (27.3%)	2635 (26.0%)	1149 (22.3%)	948 (27.7%)	
2006–2010	20,769 (32.2%)	3322 (32.8%)	1629 (31.6%)	1148 (33.5%)	
2011–2015	26,105 (40.5%)	4164 (41.1%)	2379 (46.1%)	1332 (38.9%)	
FIGO stage					**<0.001**
I	33,589 (52.1%)	6989 (69.1%)	2952 (57.2%)	2094 (61.1%)	
II	15,657 (24.3%)	1968 (19.4%)	926 (18.0%)	859 (25.1%)	
III	9329 (14.5%)	559 (5.5%)	556 (10.8%)	236 (6.9%)	
IV	5937 (9.2%)	605 (6.0%)	723 (14.0%)	239 (7.0%)	
Histology					n.a.
Squamous cell	64,512 (100%)	0	0	0	
Adenosquamous	0	0	0	3428 (100%)	
Adenocarcinoma					
Endocervical usual type	0	8194 (81.0%)	0	0	
Endometrioid	0	1927 (19.0%)	0	0	
Mucinous	0	0	617 (12.0%)	0	
Serous	0	0	311 (6.0%)	0	
Clear	0	0	543 (10.5%)	0	
NOS	0	0	3686 (71.5%)	0	
Initial treatment					**<0.001**
Surgery	38,424 (59.6%)	8637 (85.3%)	3512 (68.1%)	2781 (81.1%)	
CCRT	14,395 (22.3%)	797 (7.9%)	758 (14.7%)	368 (10.7%)	
RT only	10,461 (16.2%)	446 (4.4%)	434 (8.4%)	200 (5.8%)	
Chemotherapy only	840 (1.3%)	200 (2.0%)	246 (8.3%)	69 (2.0%)	
Others	392 (0.6%)	41 (0.4%)	27 (0.5%)	10 (0.3%)	

The number (%) or median (interquartile range) is shown. Abbreviations: FIGO, International Federation of Gynecology and Obstetrics; SCC, squamous cell carcinoma; AC, adenocarcinoma; AS, adenosquamous; CCRT, concurrent chemoradiation; RT, radiotherapy; and NOS, not otherwise specified.

**Table 2:** Multivariable analysis of cervical cancer-specific survival (*n* = 83,218).

	Survival	Univariable		Multivariable	
Characteristic	5-yr (%)	HR (95% CI)	*p-*Value	HR (95% CI)	*p-*Value
Age (years)					
≤39	86.6%	0.47 (0.43–0.50)	**<0.001**	1.11 (1.03–1.19)	**0.008**
40–49	80.6%	0.70 (0.66–0.75)	**<0.001**	1.08 (1.01–1.15)	**0.02**
50–59	73.0%	1		1	
60–69	74.4%	0.94 (0.88–1.01)	0.07	0.86 (0.81–0.92)	**<0.001**
≥70	66.0%	1.36 (1.27–1.45)	**<0.001**	1.04 (0.98–1.10)	0.23
Registry Area					
North	78.4%	0.90 (0.82–0.99)	**0.03**	1.10 (0.99–1.20)	0.051
Central	76.1%	1.00 (0.93–1.07)	0.98	1.09 (1.02–1.16)	**0.02**
East	76.2%	1		1	
West	78.2%	0.91 (0.87–0.96)	**<0.001**	0.95 (0.91–1.00)	0.055
Year at diagnosis					
2001–2005	68.7%	1		1	
2006–2010	80.4%	0.58 (0.55–0.61)	<0.001	0.63 (0.60–0.66)	**<0.001**
2011–2015	83.0%	054 (0.49–0.58)	<0.001	0.57 (0.53–0.62)	**<0.001**
FIGO stage					
I	92.6%	1		1	
II	74.1%	3.81 (0.36–4.08)	**<0.001**	3.63 (3.38–3.90)	**<0.001**
III	54.6%	8.20 (7.67–8.77)	**<0.001**	6.67 (6.11–7.25)	**<0.001**
IV	26.2%	19.7 (18.4–21.1)	**<0.001**	15.19 (14.0–16.5)	**<0.001**
Histology					
Squamous cell	78.0%	1		1	
Adenosquamous	74.1%	1.10 (0.99–1.22)	0.08	1.50 (1.35–1.66)	**<** **0.001**
Type 1 adenocarcinoma	75.4%	1.15 (1.08–1.23)	**<0.001**	1.95 (1.82–2.08)	**<0.001**
Type 2 adenocarcinoma	68.9%	1.58 (1.46–1.71)	**<0.001**	2.00 (1.84–2.15)	**<0.001**
Initial treatment					
Surgery	88.1%	1		1	
CCRT	59.8%	4.37 (4.15–4.61)	**<0.001**	1.59 (1.48–1.71)	**<0.001**
RT alone	59.0%	4.42 (4.17–4.69)	**<0.001**	1.35 (1.25–1.48)	**<0.001**
Chemotherapy alone	19.9%	15.9 (14.4–17.7)	**<0.001**	2.05 (1.89–2.23)	**<0.001**
Others	67.1%	3.92 (3.02–5.08)	**<0.001**	2.45 (2.05–3.42)	**<0.001**

Cox proportional hazard regression models for multivariable analyses. All listed covariates were entered into the final model. Significant *p*-values are emboldened. Abbreviations: FIGO, International Federation of Gynecology and Obstetrics; CCRT, concurrent chemoradiation; RT, radiotherapy; HR, hazard ratio; CI, confidence interval; and 5-yr (%), 5-year proportion.

## Supplementary Materials

The following are available online at https://www.mdpi.com/2072-6694/12/5/1251/s1, Figure S1: Cause-specific survival for adenocarcinoma subtypes based on the WHO/IECC classification and Figure S2: Cause-specific survival for histological subtypes (SEER cohort), Table S1: Classification of cervical adenocarcinoma, Table S2: Patient demographics of cervical adenocarcinomas, Table S3: Independent contributing factors for type 2 adenocarcinoma (*N* = 83,218), Table S4: Independent contributing factors for type 2 adenocarcinoma among whole adenocarcinoma cohort (*N* = 15,278), and Table S5: Clinicopathological characteristics of cervical cancer (SEER cohort).
