# Supplementary material for: Proposal of a Two-Tier System in Grouping Adenocarcinoma of the Uterine Cervix
Source: Cancers (Basel). 2020 May 15;12(5):1251. doi: 10.3390/cancers12051251 (PMC7281760; doi:10.3390/cancers12051251)
Supplement: Supplementary file 1 [file cancers-12-01251-s001.pdf]

cancers

MDPI

Supplementary Materials: Proposal of a Two-Tier System in Grouping Adenocarcinoma of the Uterine Cervix

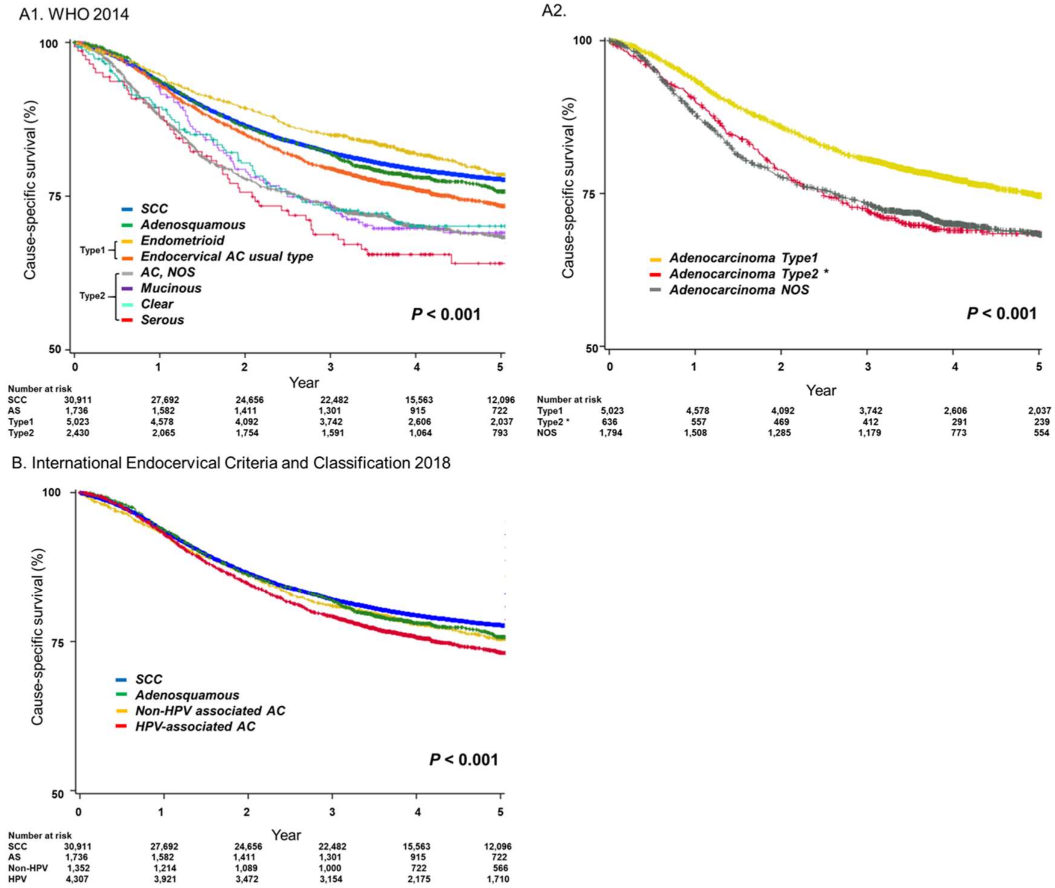

Figure S1. Cause-specific survival for adenocarcinoma subtypes based on the WHO/IECC classification. Log-rank test for *p*-value. The Y-axis was truncated to 50–100%. Survival curves were constructed for cause-specific survival of women with histological subtypes based on the WHO 2014 classification (panel **A1**) and the IECC 2018 classification (panel **B**). Among cervical adenocarcinomas based on WHO 2014 classification, validation of survival analysis was performed (panel **A2**). * Abbreviations: WHO, World Health Organization; IECC, International Endocervical Criteria and Classification; HPV, human papillomavirus; SCC, squamous cell carcinoma; AC, adenocarcinoma; AS, adenosquamous; and NOS, not otherwise specified.

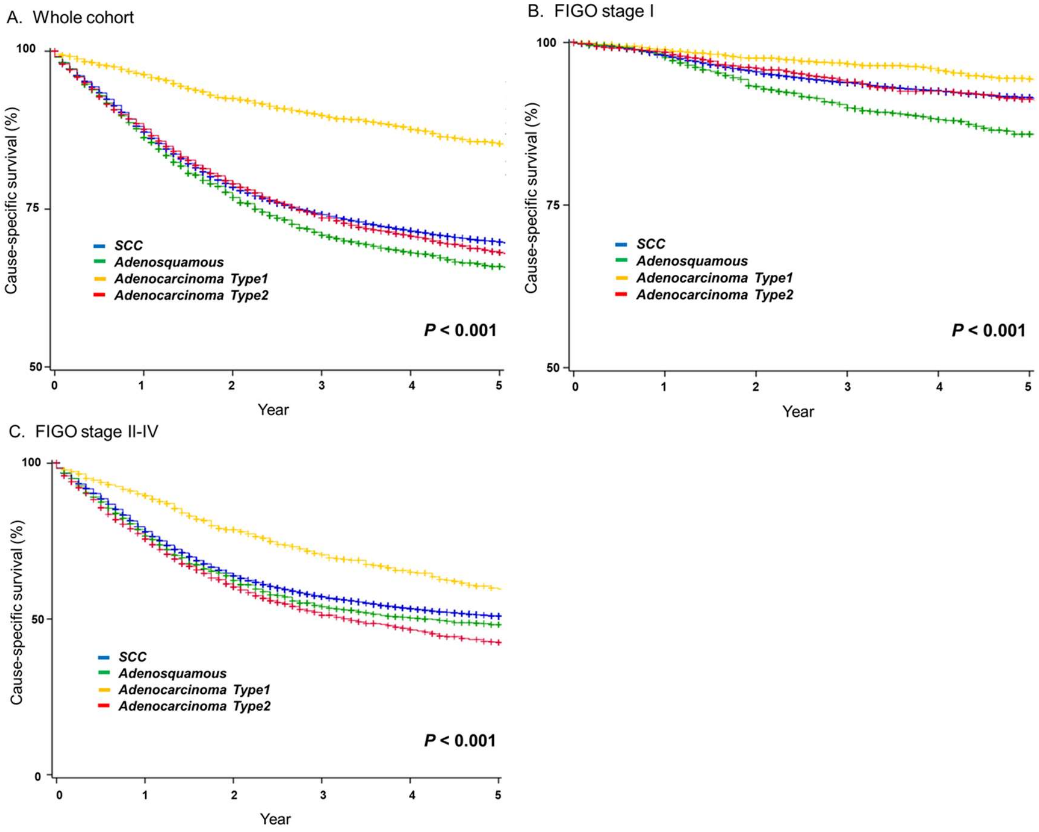

Figure S2. Cause-specific survival for histological subtypes (SEER cohort). Log-rank test for *P*-value. The Y-axis was truncated to 50%–100% for panel **A**–**B** and 0–100% for panel **C**. Survival curves were constructed for the cause-specific survival of women with whole cohort (panel **A**), women with stage I (panel **B**), and women with stage II–IV (panel **C**).

Table S1. Classification of cervical adenocarcinoma.

WHO 2014	IECC 2018	
	**HPV-Associated**	**Non-HPV-Associated**
Endocervical usual type	Endocervical usual type	
Mucinous, NOS	Mucinous, NOS	
Mucinous, gastric type		Mucinous, gastric type
Mucinous, intestinal type	Mucinous, intestinal type	
Endometrioid		Endometrioid
Clear cell		Clear cell
Serous		Serous

Adenocarcinoma classification by the WHO was compared to that by the IECC. Abbreviations: WHO, World Health Organization; IECC, International Endocervical Criteria and Classification; HPV, human papillomavirus; and NOS, not otherwise specified.

Table S2. Patient demographics of cervical adenocarcinomas.

Characteristics	AC Type 1	AC Type 2		*p*-value
		mucinous, serous, and clear cell	Adenocarcinoma NOS	
**Number**	**10,121 (66.2%)**	1,471 (9.6%)	3,686 (24.2%)	
Age (years)	49 (40–60)	54 (43–65)	53 (41-65)	**<0.001**
<40	2,326 (23.0%)	227 (15.4%)	746 (20.2%)	
40–49	2,971 (29.4%)	372 (25.3%)	949 (25.7%)	
50–59	2,282(22.5%)	303 (20.6%)	728 (19.8%)	
60–69	1,517 (15.0%)	320 (21.8%)	614 (16.7%)	
≥70	1,025(10.1%)	249 (16.8%)	649 (17.6%)	
Registry area				**<0.001**
North	6,61 (6.5%)	144 (9.8%)	240 (6.5%)	
Central	1,615 (16.0%)	220 (15.0%)	584 (15.8%)	
East	3,784 (37.4%)	487 (33.1%)	1,414 (38.4%)	
West	4,061 (40.1%)	620 (42.1%)	1,448 (39.3%)	
Year at diagnosis				**<0.001**
2001–2005	2,635 (26.0%)	348 (23.7%)	801 (21.7%)	
2006–2010	3,322 (32.8%)	415 (28.2%)	1,214 (32.9%)	
2011–2015	4,164 (41.1%)	708 (48.1%)	1,671 (45.3%)	
FIGO stage				**<0.001**
I	6,989 (69.1%)	924 (62.8%)	2,028 (55.0%)	
II	1,968 (19.4%)	276 (18.8%)	650 (17.6%)	
III	559 (5.5%)	116 (7.9%)	440 (11.9%)	
IV	605 (6.0%)	155 (10.5%)	568 (15.4%)	
Histology				n.a.
Endocervical usual type	8,194 (81.0%)	0	0	
Endometrioid	1,927 (19.0%)	0	0	
Mucinous	0	617 (41.4%)	0	
Serous	0	311 (20.8%)	0	
Clear	0	543 (36.4%)	0	
Adenocarcinoma, NOS	0	0	3,686 (100%)	
Initial treatment				**<0.001**
Surgery	8,637 (85.3%)	1,183 (80.4%)	2,329 (63.2%)	
CCRT	797 (7.9%)	131 (8.9%)	626 (17.0%)	
RT only	446 (4.4%)	94 (6.3%)	368 (10.0%)	
Chemotherapy only	200 (2.0%)	61 (4.1%)	338 (9.2%)	
Others	41 (0.4%)	2 (0.1%)	25 (0.7%)	

The number (%) or median (interquartile range) is shown. Abbreviations: FIGO, International Federation of Gynecology and Obstetrics; AC, adenocarcinoma; NOS, not otherwise specified; CCRT, concurrent chemoradiation; RT, radiotherapy; and n.a., not available.

Table S3. Independent contributing factors for type 2 adenocarcinoma (N = 83,218).

Characteristic	Whole Cohort Number (%)	Adenocarcinoma Type 2	Adjusted OR (95% CI)	*p*-value
Age (years)				
<40	19,333 (23.2%)	973 (18.9%)	0.71 (0.65-0.78)	**<0.001**
40–49	19,722 (23.7%)	1,321 (25.6%)	0.98 (0.90-1.07)	0.67
50–59	16,123 (19.4%)	1,031 (20.0%)	1	
60–69	14,196 (17.1%)	934 (18.1%)	1.07 (0.97-1.17)	0.18
≥70	13,844 (16.6%)	898 (17.4%)	1.32 (1.19-1.45)	**<0.001**
Registry area				
North	5,655 (6.8%)	384 (7.4%)	1.04 (0.93–1.16)	0.53
Central	12,371 (14.9%)	804 (15.6%)	0.99 (0.91–1.09)	0.92
East	29,751 (35.8%)	1,901 (36.9%)	1	
West	35,441 (42.6%)	2,068 (40.1%)	0.89 (0.83–0.95)	**<0.001**
Year at diagnosis				
2001–2005	22,370 (26.9%)	1,149 (22.3%)	1	
2006–2010	26,868 (32.3%)	1,629 (31.6%)	1.21 (1.12–1.30)	**<0.001**
2011–2015	33,980 (40.8%)	2,379 (46.1%)	1.36 (1.26–1.46)	**<0.001**
FIGO stage				
I	45,624 (54.8%)	2,952 (57.2%)	1	
II	19,410 (23.3%)	926 (18.0%)	0.81 (0.74–0.87)	**<0.001**
III	10,680 (12.8%)	556 (10.8%)	1.15 (1.02–1.30)	**0.03**
IV	7,504 (9.1%)	723 (14.0%)	1.88 (1.67–2.11)	**<0.001**
Initial treatment				
Surgery	53,354 (64.1%)	3,512 (68.1%)	2.11 (1.89–2.36)	**<0.001**
CCRT	16,318 (19.6%)	758 (14.7%)	1	
RT only	11,541 (13.9%)	434 (8.4%)	1.09 (0.96–1.24)	0.17
Chemotherapy only	1,355 (1.6%)	246 (8.3%)	2.33 (2.04–2.66)	**<0.001**
Others	470 (0.6%)	27 (0.5%)	1.34 (0.90–1.99)	0.15

Number (%) is shown. Percentages are shown per column. A binary logistic regression model was used for multivariate analysis (all significant covariates in the univariate analysis were entered in final model). Significant *P*-values are emboldened. Abbreviations: FIGO, International Federation of Gynecology and Obstetrics; CCRT, concurrent chemoradiation; RT, radiotherapy; No., number; OR, odds ratio; and CI, confidence interval.

Table S4. Independent contributing factors for type 2 adenocarcinoma among whole adenocarcinoma cohort (N = 15,278).

Characteristic	Whole AC Cohort Number (%)	Adenocarcinoma Type 2	Adjusted OR (95% CI)	*p*-value
Age (years)				
<40	3,299 (21.6%)	973 (18.9%)	1.01 (0.91-1.12)	0.85
40–49	4,292 (28.1%)	1,321 (25.6%)	0.93 (0.83-1.03)	0.18
50–59	3,313 (21.7%)	1,031 (20.0%)	1	
60–69	2,451 (16.0%)	934 (18.1%)	1.17 (1.04-1.31)	**0.008**
≥70	1,923 (12.6%)	898 (17.4%)	1.32 (1.16-1.50)	**<0.001**
Registry area				
North	1,045 (6.8%)	384 (7.4%)	1.20 (1.04-1.38)	**0.012**
Central	2,419 (15.8%)	804 (15.6%)	0.99 (0.90-1.10)	0.89
East	5,685 (37.2%)	1,901 (36.9%)	1	
West	6,129 (40.1%)	2,068 (40.1%)	1.04 (0.96-1.13)	0.32
Year at diagnosis				
2001–2005	3,784 (24.8%)	1,149 (22.3%)	1	
2006–2010	4,951 (32.4%)	1,629 (31.6%)	1.10 (1.01-1.21)	**0.038**
2011–2015	6,543 (42.8%)	2,379 (46.1%)	1.25 (1.14-1.37)	**<0.001**
FIGO stage				
I	9,941 (65.1%)	2,952 (57.2%)	1	
II	2,894 (18.9%)	926 (18.0%)	0.89 (0.81-0.98)	**0.02**
III	1,115 (7.3%)	556 (10.8%)	1.24 (1.06-1.46)	**0.007**
IV	1,328 (8.7%)	723 (14.0%)	1.54 (1.32-1.78)	**<0.001**
Initial treatment				
Surgery	12,148 (79.5%)	3,512 (68.1%)	1	
CCRT	1,447 (9.5%)	758 (14.7%)	2.10 (1.83-2.42)	**<0.001**
RT only	817 (5.3%)	434 (8.4%)	2.16 (1.83-2.55)	**<0.001**
Chemotherapy only	798 (5.2%)	246 (8.3%)	2.04 (1.71-2.44)	**<0.001**
Others	68 (0.4%)	27 (0.5%)	1.26 (0.76-2.07)	0.37

Number (%) is shown. Percentages are shown per column. A binary logistic regression model was used for multivariate analysis (all significant covariates were entered in final model). Significant *P*-values are emboldened. Abbreviations: FIGO, International Federation of Gynecology and Obstetrics; CCRT, concurrent chemoradiation; RT, radiotherapy; No., number; OR, odds ratio; and CI, confidence interval.

Table S5. Clinicopathological characteristics of cervical cancer (SEER cohort).

Characteristics	SCC	AC Type 1	AC Type 2	AS	*p*-value
Number	34,223 (85.3%)	2,160 (5.4%)	1,658 (4.1%)	1,925 (4.8%)	
Age (years)	49 (39–61)	46 (38–56)	50 (40–63)	46 (38–57)	**<0.001**
<40	8,862 (25.9%)	617 (28.6%)	366 (22.1%)	565 (29.4%)	
40–49	8,845 (25.8%)	676 (31.3%)	419 (25.3%)	569 (29.6%)	
50–59	7,254 (21.2%)	457 (21.2%)	340 (20.5%)	381 (19.8%)	
60–69	4,845 (14.2%)	253 (11.7%)	247 (14.9%)	234 (12.2%)	
≥70	4,417 (12.9%)	157 (7.3%)	286 (17.2%)	176 (9.1%)	
Race/ethnicity					**<0.001**
White	17,502 (51.1%)	1,414 (65.5%)	1,020 (61.5%)	1,008 (52.4%)	
Black	5,548 (16.2%)	130 (6.0%)	140 (8.4%)	220 (11.4%)	
Hispanic	7,555 (22.1%)	392 (18.1%)	287 (17.3%)	461 (23.9%)	
Asian	2,662 (7.8%)	165 (7.6%)	171 (10.3%)	190 (9.9%)	
Others	956 (2.8%)	59 (2.7%)	40 (2.4%)	46 (2.4%)	
Registry area					**<0.001**
Central	7,254 (21.2%)	410 (19.0%)	295 (17.8%)	316 (16.4%)	
East	9,388 (27.4%)	560 (25.9%)	454 (27.4%)	474 (24.6%)	
West	17,581 (51.4%)	1,190 (55.1%)	909 (54.8%)	1,135 (59.0%)	
Year at diagnosis					**<0.001**
2001–2005	12,098 (35.4%)	578 (26.8%)	539 (32.5%)	736 (38.2%)	
2006–2010	11,361 (33.2%)	708 (32.8%)	551 (33.2%)	649 (33.7%)	
2011–2015	10,764 (31.5%)	874 (40.5%)	568 (34.3%)	540 (28.1%)	
AJCC 7th stage					**<0.001**
I	15,454 (46.6%)	1,552 (73.6%)	804 (48.5%)	894 (47.6%)	
II	5,508 (16.6%)	185 (8.8%)	223 (13.4%)	261 (13.9%)	
III	7,789 (23.5%)	229 (10.9%)	323 (19.5%)	429 (22.8%)	
IV	4,401 (13.3%)	142 (6.7%)	266 (16.0%)	294 (15.7%)	
Squamous cell	34,223 (100%)	0	0	0	n.a.
Adenosquamous	0	0	0	1,925 (100%)	
Adenocarcinoma					
Endocervical usual type	0	12,58 (58.2%)	0	0	
Endometrioid	0	902 (41.8%)	0	0	
Mucinous	0	0	875 (52.8%)	0	
Serous	0	0	352 (21.2%)	0	
Clear	0	0	431 (26.0%)	0	
Initial treatment					**<0.001**
Surgery	17,967 (52.5%)	1,813 (83.9%)	1,179 (71.1%)	12,94 (67.2%)	
CCRT	10,876 (31.8%)	185 (8.6%)	274 (16.5%)	424 (22.0%)	
RT only	2,398 (7.0%)	51 (2.4%)	67 (4.0%)	84 (4.4%)	
Chemotherapy only	616 (1.8%)	17 (0.8%)	34 (2.1%)	38 (2.0%)	
Others	2,366 (6.9%)	94 (4.4%)	104 (6.3%)	85 (4.4%)	

The number (%) or median (interquartile range) is shown. Abbreviations: SEER, Surveillance, Epidemiology, and End Results program; and CCRT, concurrent chemoradiation; n.a, not available.
